# Draft Genome Sequences of Three *Capnocytophaga cynodegmi* Strains Isolated from the Oral Cavity of Healthy Dogs

**DOI:** 10.1128/genomeA.00200-15

**Published:** 2015-05-28

**Authors:** Pablo Manfredi, Francesco Renzi, Guy R. Cornelis

**Affiliations:** a Biozentrum der Universität Basel, Basel, Switzerland; b University of Namur, Namur, Belgium

## Abstract

Here, we present the draft genome sequences of three strains of *Capnocytophaga cynodegmi*. In contrast to the very close relationship among them, *C. cynodegmi* and *Capnocytophaga canimorsus* differ dramatically in terms of virulence in humans. Comparative genomics provided some understanding on how *Capnocytophaga* species may switch from being dog commensals to human pathogens.

## GENOME ANNOUNCEMENT

*Capnocytophaga cynodegmi* (formerly CDC dysgonic fermenter-2-like) is a common oral commensal of dogs and cats, with prevalence rates as high as 86% and 84%, respectively (1). It belongs to the *Bacteroidetes* phylum, where it is very closely related to *Capnocytophaga canimorsus*, another commensal of the dog mouth (2, 3). Together, the two species display a significant number of features that differentiate them from other members of the *Capnocytophaga* genus (4). Beside subtle differences in their 16S rRNA gene sequences (4), *C. cynodegmi* strains can be differentiated from *C. canimorsus* strains by the light-yellow color displayed by colonies grown on sheep blood agar and by the capacity to ferment sucrose, raffinose, inulin, and melibiose (4). Contrary to *C. canimorsus*, *C. cynodegmi* is not a severe and sepsis-causing human pathogen, although some cases of human infections have been reported to consist mostly of wound or corneal infections (4, 5–8).

Three strains of *C. cynodegmi*, Ccy74, Ccyn2B, and Ccyn_ATCC 49044, were isolated from canine oral swabs and identified by 16S RNA sequencing (4, 9). The strains were selected as dispersed representatives of the species *C. cynodegmi*, according to 16S rRNA phylogenetics and limited phenotyping (9). Genomic DNA was extracted using the Genomic-tip 500/G DNA extraction kit (catalog no. 10262; Qiagen), according to the manufacturer’s instructions, followed by an additional phenol-chloroform purification step. Sequencing was performed at LGC Genomics, Berlin, Germany, on one Illumina HiSeq 2000 channel and generated approximatively 10.8 ± 1.0 million 100-bp single reads per strain. *De novo* assembly was performed with Velvet, with optimized parameters (10). On average, draft assemblies accounted for 2.69 ± 0.01 Mb for 90 (Ccy74), 67 (Ccyn2B), and 111 (Ccyn_ATCC 49044) contigs. Genome metrics and automated annotation were conducted at the LABGeM, France Génomique (11). The G+C content (34.40% ± 0.01%) is lower than that of the closely related *C. canimorsus* (36.16% ± 0.08%). Each genome contains a fairly similar number of coding sequences (2,484 ± 18). Ccyn2B exhibits significantly more strain-specific coding sequences (CDSs) (350) than those in Ccy74 (75) and Ccyn_ATCC 49044 (72). The *C. cynodegmi* core genome is composed of 1,910 families of orthologs, of which 341 are specific to *C. cynodegmi* compared to seven genomes of *C. canimorsus* (2, 3, 12). While 253 clusters of orthologs were of unknown function, genes involved in aromatic amino acid synthesis (the complete l-tryptophan synthesis pathway from chorismate, 5 genes), glycan chain foraging (a complete polysaccharide utilization locus, 8 genes) (13), oxidative respiration and oxidative stress resistance (5 genes), and lipopolysaccharide (LPS) and polysaccharide biosynthesis (5 genes) formed the major functional clusters of the species-exclusive core genome. With respect to iron acquisition, a homolog to the heme-binding HmuY protein (14) was found in each of the three *C. cynodegmi* genomes, in addition to a locus encoding the iron capture system of *Bacteroidetes* (15).

### Nucleotide sequence accession numbers. 

These whole-genome shotgun projects have been deposited in ENA under the accession numbers CDOD00000000 (Ccyn2B), CDOG00000000 (Ccy74), and CDOF00000000 (Ccyn_ATCC 49044). The versions described in this paper are the initial versions.
